# Development and Validation of a Simultaneous RP-HPLC Method for the Estimation of Ferrous Fumarate and Ascorbic Acid for Pharmaceutical Quality Control

**DOI:** 10.1021/acsomega.6c02561

**Published:** 2026-08-18

**Authors:** Vinay Kumar, Muralidhar Pisay, Anoushka Mukharya, Komal Rana, Sanjay Bharati, Muddukrishna Badamane Sathyanarayana, Srinivas Mutalik

**Affiliations:** † Department of Pharmaceutics, Manipal College of Pharmaceutical Sciences, 76793 Manipal Academy of Higher Education, Manipal 576104, Karnataka, India; ‡ Manipal – Government of Karnataka Bioincubator, Advanced Research Centre, Manipal Academy of Higher Education, Manipal 576104, Karnataka, India; § Department of Nuclear Medicine, Manipal College of Health Professions, Manipal Academy of Higher Education, Manipal 576104, Karnataka, India; ∥ Department of Pharmaceutical Quality Assurance, Manipal College of Pharmaceutical Sciences, Manipal Academy of Higher Education, Manipal 576104, Karnataka, India

## Abstract

Our aim was to develop an accurate, precise, and reliable RP-HPLC method for estimating ferrous fumarate and ascorbic acid in pro-liposomes. The aim was to make the analytical method simple, environmentally friendly, and accurate. To measure ferrous fumarate (FF) and ascorbic acid (AA), an isocratic method was used in an HPLC, with 220 and 245 nm detection, using phosphate buffer (pH 3.0) and methanol (95:5). It was found that AA and FF were retained for 6.4 and 8.3 min, respectively. A linear relationship was observed between 0.1 and 50 μg/mL for both ferrous fumarate (*r* ^2^ = 0.994) and ascorbic acid (*r* ^2^ = 0.999). The resolution between the two peaks ≥2, tailing factor (TF) ≤2, and theoretical plate count >2000 were evaluated and recorded as system suitability parameters. The LOD and LOQ for FF were calculated to be 0.52 and 1.58 μg/mL, respectively, whereas the same for AA were calculated to be 0.17 and 0.53 μg/mL, respectively. The validation method was tested under various conditions, including flow rate, injection volume, column temperature, and mobile phase ratio. Additionally, the optimized method was successfully used to estimate the entrapment efficiency for FF and AA in the developed pro-liposomes, which were found to be 92 and 65%, respectively.

## Graphical abstract

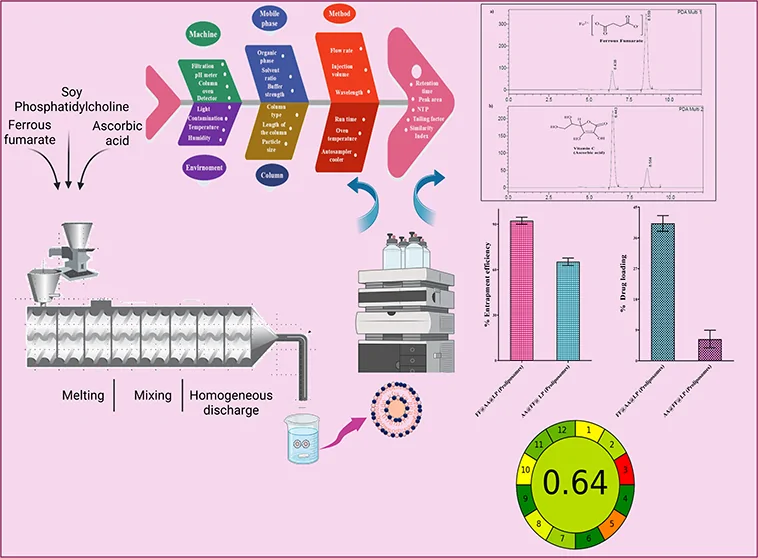

## 1 Introduction

Iron deficiency anemia ranks among the most prevalent nutritional disorders globally, with a greater proportion mostly seen in females in their reproductive years and in infants.^1^ An important micronutrient, iron is crucial for controlling oxidative metabolism, cellular immunological response, and erythropoiesis.^2^ Intestinal absorption of iron is strictly regulated since the human body does not have an active mechanism for excreting iron.^3^ It requires only a small amount of iron (1 to 2 mg) to compensate for physiological losses, including menstruation, minor bleeding, and intestinal absorption.^4^ During periods of rapid growth or pregnancy or when blood loss occurs, this requirement increases rapidly. Ferrous fumarate (FF) is extensively recommended as an oral iron supplement and serves as an important source of elemental iron due to its high iron content and adequate bioavailability.^2^ Meanwhile, FF exhibits poor flow properties, which often lead to compression difficulties, and it also possesses a characteristic metallic taste and odor.^5,6^ Although FF exhibits greater efficacy, its therapeutic use is often limited by gastrointestinal side effects compared to other iron formulations.^7^ FF is soluble only in acidic conditions, including gastric fluid, and is completely insoluble at neutral pH.^8^ The poor dissolution and minimal bioavailability of FF are due to its restricted aqueous solubility.^9^ The ferric form of iron is more prone to oxidation in a strongly acidic environment. Ferrous iron is primarily absorbed in the duodenum after reduction from ferric iron, while ferric iron is the more stable form in the stomach.^10,11^

Ascorbic acid (Vitamin C), which is naturally found in citrus fruits in abundance, displays excellent antioxidant potential and may help eliminate free radicals associated with iron overload during supplementation.^12,13^ Ascorbic acid boosts iron absorption through chelation during supplementation by increasing bioavailability.^14^ FF appears as a brownish amorphous solid, and AA appears as a white crystalline solid. Their molecular weights are 169.9 and 176.12 g/mol, with melting points around 280 and 190 °C for FF and AA, respectively. AA has limitless potential in the arenas of pharmaceuticals, food, and cosmetics by virtue of its antioxidant potential and cost-effectiveness.^15–17^

The simultaneous, accurate, and reproducible measurement of analytes such as FF and AA in various combination formulations presents substantial analytical challenges, primarily due to AA’s instability at neutral and basic pH levels. Reflecting this need, a robust chromatographic method is required that is sensitive, selective, reproducible, and has an edge over traditional techniques like colorimetric or titrimetric methods. The estimation of AA by HPLC has been described in several ways, but the simultaneous estimation of FF and AA has hardly been explored. For instance, Chebrolu et al. estimated AA using a Thermo Finnigan HPLC system with ammonium dihydrogen phosphate buffer (pH 2.6) under isocratic elution at 254 nm;^18^ whereas Klimczak and Gliszczyńska-Świgło compared HPLC and UPLC techniques employing C18 columns and gradient elution at 245 nm.^19^ Similarly, Kand’ár et al. proposed an HPLC method that uses 2.5% ethanol and sodium dihydrogen phosphate buffer (pH 4.6) as the mobile phase.^20^ In another investigation, Biltekin et al. studied an HPLC method established for the rapid and simultaneous analysis of vitamin C and oxalic acid in fresh beverages. A phosphate buffer at pH 2.3 was used as the mobile phase on a C18 column at a column temperature of 25 °C for the detection of vitamin C at 245 nm, and oxalic acid at 205 nm.^21^

Despite recent progress, optimizing methods to achieve simultaneous retention, resolution, and reproducibility at physiologically relevant pH levels remains a significant analytical hurdle. Given these challenges, there is a clear need for a validated, economical, and reliable analytical method to simultaneously estimate ferrous fumarate (FF) and ascorbic acid (AA). Moreover, pH maintenance is essential when developing and validating methods for simultaneously estimating ferrous fumarate and ascorbic acid. Thus, the present manuscript aims to establish and validate a robust, sustainable, isocratic RP-HPLC method in conformity with the ICH Q2 (R1) recommendations. The method provides short retention times and good resolution, accuracy and precision for routine simultaneous estimation of ferrous fumarate (FF) and ascorbic acid (AA) in encapsulated formulations, making it suitable for pharmaceutical quality control. Additionally, this method uses minimal organic phase, short run times, and a higher Green Analytical Chemistry (GAC) Score, a metric used to evaluate the overall greenness or environmental impact of an analytical method, making it more sustainable and less hazardous. The developed method provides excellent chromatographic performance with reduced organic solvent consumption and simplified sample processing in accordance with green analytical chemistry principles (AGREE, GAPI, and BAGI). In addition, the method remains highly effective while maintaining robust analytical performance because the solvent usage is reduced, lowering operational costs and minimizing environmental impact.

Furthermore, this work explores the coloading of FF and AA into pro-liposomes and evaluates key formulation parameters, including drug loading and entrapment efficiency, thereby providing a proof of concept for their combined delivery in the management of iron deficiency anemia.

## 2 Methodology

### 2.1 Materials

Ferrous fumarate (FF; Cat. No. F1378) and ascorbic acid (AA; Cat. No. 01549) were procured from Otto Chemie Pvt. Ltd. (Mumbai, India), and Loba Chemie Pvt. Ltd. (Mumbai, India), respectively. HPLC-grade methanol (Cat. No. 106035) and orthophosphoric acid (85%, Cat. No. 100573) were obtained from Merck, Mumbai, India. Phosphate buffer tablets (Cat. No. 5247D) were purchased from Loba Chemie Pvt. Ltd., Mumbai, India. Syringe filters (Cat. No. SFNY25RB) and membrane filters Nylon (0.22 μm) (Cat. No. 170047R) for vacuum HPLC assembly were obtained from Axiva Sichem Pvt. Ltd., New Delhi, India. HPLC-grade chemicals and methanol were used without further purification.

### 2.2 Instrumentation and Apparatus

Shimadzu LC-2010 CHT systems include quaternary low-pressure gradient pumps, degassers, column ovens, autosamplers, and a photodiode array (PDA) detector. In the current work, the chromatographic method was further modified and enhanced from the findings of a previously published research.^22^ For the separation of ferrous fumarate and ascorbic acid, a reverse-phase C18 column (Sr., No. #5701–0059; Hyperclone ODS; Phenomenex, Hyderabad, India; 250 × 4.6 mm; particle size of 5 μm) was used. The output chromatograms were optimized using the LC Solution 5.57 application, which includes acquisition time, column oven temperature, flow rate, and data acquisition. Method validation included linearity, range, specificity, accuracy, precision, and robustness. A vacuum-assisted glass filtration assembly was used to filter the phosphate buffer (pH 3.0) using a 0.22-μm nylon membrane filter. Simultaneously, an ultrasonic bath was utilized for sonication and assisted with degassing for 15 min. Milli Q water (Millipore Corporation) was used for mobile phase preparation and, wherever applicable, throughout the study. A pH meter from Systronics, Gujarat, India, was used to regulate the pH of the mobile phase.

### 2.3 Method Development

#### 2.3.1 Chromatographic Conditions

In this study, Shimadzu LC-2010 CHT system combined with UV and PDA detectors was used to develop HPLC method. This HPLC system was configured to inject 20 μL of solution, maintain a column temperature of 30 °C, have an acquisition time of 12 min per run, and maintain a sample cooler temperature of 15 °C. The isocratic flow rate was optimized and programmed for 0.6 mL/min using mobile phases consisting of phosphate buffer (pH 3.0) and methanol. FF and AA were detected at wavelengths of 220 and 245 nm, respectively. Different combinations of HPLC-grade solvents, including methanol, acetonitrile, and buffers, were evaluated to improve the HPLC analytical method. The combination of phosphate buffer and methanol (phosphate buffer pH 3.0: methanol, 95:5 v/v) yielded superior results compared with other solvent combinations. This mobile phase combination was therefore selected for further optimization and development of the method.

#### 2.3.2 Preparation of Calibration Standard and Quality Control Samples

Ferrous fumarate (FF, 5 mg) and ascorbic acid (AA, 5 mg) were accurately weighed and dissolved in 50 mL of mobile phase (phosphate buffer pH 3.0: methanol, 95:5 v/v) separately in 50 mL volumetric flasks. The volumetric flasks were sonicated for 40 min or until FF was completely dissolved, while avoiding overheating of the sample. An aliquot of 5 mL from each solution containing FF and AA was transferred into a separate 50 mL volumetric flask and diluted to the mark with mobile phase to prepare primary stock solutions (100 μg/mL). This primary stock solution was further diluted to obtain a final stock solution (50 μg/mL). Working solutions of ferrous fumarate (FF) and ascorbic acid (AA) were prepared by serial dilution of the final stock solution (50 μg/mL) in the mobile phase to create a 6-point dilution series, ranging from 50 to 0.1 μg/mL, to determine the linearity of the optimized method.^23^ Calibration standards were prepared at concentrations of 0.1, 10, 20, 30, 40, and 50 μg/mL, while quality control (QC) samples were prepared at 4, 5, and 6 μg/mL (low, middle, and high concentrations, respectively) at 1.5 mL sample volume each (Table 1).

**Table 1:** Preparation of Standard Calibration Solutions and Dilution Volumes (1.5 mL Each)

standard solutions	volume taken from the 50 μg/mL stock solution (μL)	mobile phase added (μL)	prepared final conc. (μg/mL)
calibration standards	3	1497	0.1
	300	1200	10
	600	900	20
	900	600	30
	1200	300	40
	1500	0	50
quality control standards	120	1380	4
	150	1350	5
	180	1320	6

Serial dilution of FF and AA was further quantified using the optimized HPLC method to determine the linearity of both compounds separately. All samples were filtered through a 0.22 μm membrane filter prior to injection using an autoinjector for the standard calibration plot.

### 2.4 Method Validation

#### 2.4.1 Linearity and Range

A 6-point dilution series was prepared, ranging from 50 to 0.1 μg/mL to determine the linearity of the optimized method (Table 1).^23^ Serial dilution of the two analytes was further quantified using the optimized HPLC method to determine the linearity of both compounds separately. The correlation coefficient (*r* ^2^) value was computed. *r* ^2^ values close to 1.0 indicate a highly linear relationship and excellent predictive power, while *r* ^2^ values close to 0 indicate no linear relationship.^24^

#### 2.4.2 System Suitability Test and Sensitivity (LOQ and LOD)

As a first step in developing HPLC-based analytical methods, the System Suitability examination is essential. A standard stock solution of FF and AA in mobile phase was prepared at a concentration of 4 μg/mL (*n* = 6) to optimize the chromatographic conditions.^21,25^ Both samples were analyzed using RP-HPLC, and parameters including retention time (RT), tailing factor, resolution, and theoretical plate number (NTP) were determined. According to the United States Pharmacopoeia (USP) Chapter 621, the following acceptance criteria were applied: NTP ≥ 2000, tailing factor ≤2.0, and RT reproducibility within ± 10% of the principal peak obtained under optimized conditions. The peak purity index, limit of quantification (LOQ), and limit of detection (LOD) of the system were evaluated to confirm its suitability. As part of the validation process, ICH Q2­[R1] was incorporated. For LOD and LOQ calculations, a total of 6 dilutions were considered. These experimental readings were computed from the standard deviation of the sample (σ) and the slope of the standard plot (S). Equations 1 and 2 are presented for determining LOD and LOQ.^26^

1 $$\mathrm{LOD}=3.3^{*}\sigma /S$$

2 $$\mathrm{LOQ}=10^{*}\sigma /S$$

#### 2.4.3 Accuracy

Accuracy reflects how closely the measured value aligns with the accepted true value. As part of the accuracy assessment, three concentration levels around the midconcentration (5 μg/mL)^27^ were analyzed: lower quality control (LQC) at 80%, medium quality control (MQC) at 100%, and higher quality control (HQC) at 120% of the target concentration. To prepare quality control samples for both ferrous fumarate (FF) and ascorbic acid (AA), 120 μL, 150 μL, and 180 μL aliquots of 50 μg/mL stock solutions were diluted up to 1.5 mL with mobile phase (PBS: methanol, 95:5%) to achieve final concentrations of 4 μg/mL (LQC), 5 μg/mL (MQC), and 6 μg/mL (HQC) (Table 1). Each concentration was prepared and analyzed in triplicates (*n* = 3), and all samples were run on the same day. The accuracy of the optimized method was verified, and the absolute recovery percentage was computed using eq 3.^19^

3 %Recovery=Actual⁢concentrationrecovered/Theoretical⁢concentration×100

#### 2.4.4 Precision

Precision, defined as the closeness of agreement between replicate measurements of the same FF and AA samples under specified conditions, regardless of how close they are to the true value (ICH Q2­[R1]). The study assessed intraday and interday variations.^28^ The relative standard deviation (RSD) is calculated by performing triplicate measurements on each of the three samples (4, 5, and 6 μg/mL) and comparing the results with the standard to assess precision. Intraday precision was demonstrated within a single day, under same conditions, indicating outcomes with an overall RSD within acceptable limits. Repeatability expresses the closeness of agreement between the results of successive measurements of the same sample obtained under the same conditions over a short time interval. To achieve intermediate (interday) precision, samples are performed on different days under same laboratory conditions. In each measurement, a maximum RSD of 2.0% was used as the acceptance criterion.

#### 2.4.5 Robustness

In HPLC settings, minor internal adjustments were examined to determine whether the method was robust.^29^ The robustness of the optimized method was assessed by deliberately varying the chromatographic configurations. QC standard samples (*n* = 3) were estimated for changes in ratios of mobile phase (5 ± 2%; Methanol), flow rate (0.6 ± 0.1 mL min^–1^) and column temperature (30 ± 2 °C). The % RSD was quantified, and the effects on peak area and shift in RT were noted.

#### 2.4.6 Specificity

Specificity refers to the accuracy of the analytical procedure when ferrous fumarate or ascorbic acid is present in the sample, along with other components, such as blank matrix, mobile phase, degradation products, or excipients.

#### 2.4.7 Forced Degradation Study

An evaluation of the optimized method was performed using a forced degradation study to assess its ability to quantify FF and AA simultaneously. Various stress conditions were applied to FF and AA in accordance with ICH Q1A­(R2). In the acidic, alkaline, and neutral hydrolysis, 0.1 N HCl, 0.1 N NaOH, and 0.6% H_2_O_2_ were used, respectively. For analysis, the exposed FF and AA samples were diluted with the mobile phase and run using the optimized HPLC method.

### 2.5 Applications of Established Method

FF and AA loaded pro-liposomal formulation was prepared, characterized and further analyzed using the above optimized HPLC simultaneous estimation method.

#### 2.5.1 Preparation of Pro-Liposomes

In a previous study, solid lipid nanoparticles were prepared using hot-melt extrusion (HME) technique.^30^ In this study, we attempted to prepare pro-liposomes by following HME technique. In this method, all excipients, including soybean phosphatidylcholine (SPC; ∼2500 mg), cholesterol (∼700 mg), Tween 80 (∼100 mg), ferrous fumarate (FF; ∼500 mg), and ascorbic acid (AA; ∼1000 mg), were weighed and thoroughly mixed using a mortar and pestle. The twin-screw extrusion process involved setting the temperatures of the extruder barrels in four zones: B1 at 40 °C, B2 at 70 °C, B3 at 110 °C, and B4 at 40 °C, with a screw speed of 110 rpm.^30^ After extrusion, the pro-liposomes were homogenized to reduce the particle size effectively. The prepared formulation was further lyophilized for characterization such as particle size, ζ-potential, polydispersity index, entrapment efficiency percentage, drug loading percentage, and drug release kinetics.

#### 2.5.2 Entrapment Efficiency (%) and Drug Loading (%)

The entrapment efficiency (%) and drug loading (%) of FF and AA were determined using an earlier reported centrifugation method with minute modifications.^31^ Briefly, a 1 mL aliquot was centrifuged for 30 min at 10,000 rpm to separate free and encapsulated drugs. The supernatant was separated without without disturbing the pellet, diluted with mobile phase, and analyzed via validated HPLC to quantify FF and AA contents. The equations below were used for computing entrapment efficiency (%) and drug loading (%).^32^

4 %Entrapment efficiency=Theoretical⁢drug−unentrapped⁢druginsupernatant/Theoretical⁢drug×100

5 %Drug loading=Theoreticaldrug−unentrapped⁢drug⁢inthesupernatant/weightofnanocarrier×100

#### 2.5.3 *In Vitro* Drug Release Study

The dialysis bag method employed a membrane with a 15 kDa molecular weight cutoff for the drug release experiment.^33,34^ The dialysis membrane was activated by soaking in 20 mL of Milli-Q water for overnight prior to the experiment. After activation, the dialysis bag was loaded with 2 mg of the pure drugs FF and AA, and their respective equivalent pro-liposomal formulations (FF@AA@LP and AA@FF@LP). The sample-loaded dialysis pockets were then placed inside separate beakers containing 50 mL release medium (0.1 N HCl; pH 1.2) and incubated for 2 h. Then, the dialysis pockets were transferred to phosphate-buffered saline (PBS) of pH 4.5 for an additional 1 hy. A constant temperature of 37 °C was maintained with 150 ± 5 rpm stirring during the experiment. Fresh medium was substituted with 1 mL aliquots of the release medium at predetermined time intervals. The cumulative drug release (%) was quantified using a validated RP-HPLC method, with detection wavelengths set at 220 and 245 nm for FF and AA, respectively.^35^ FF@AA@LP and AA@FF@LP terms were used to denote FF and AA, respectively, in the pro-liposomes in Entrapment Efficiency (%), Drug Loading and In Vitro Drug Release Study.

### 2.6 Analysis of Data

#### 2.6.1 Statistical Analysis

Statistically, the experimental data were represented as mean ± standard deviation. The Microsoft Excel worksheet was used to calculate the mean, standard deviation (SD), and % RSD. All graphs, including calibration curve, *in vitro* drug release profiles were prepared using GraphPad Prism v8.0.

#### 2.6.2 Assessment of Greenness

AGREE (Analytical GREEnness Metric) is a software tool that provides both visual and quantitative measurements of analytical greenness, which was used to assess the environmental sustainability of RP-HPLC. Green Analytical Chemistry’s 12 principles form the evaluation framework, with parameters scored proportionally to their impact. Overall performance appears as an aggregated numerical score with a corresponding circular pictogram for simplified result comprehension. Systematic eco-friendliness analysis offers a basis for comparing method performance with alternative analytical approaches. A total greenness score is displayed in the center of an AGREE visualization, surrounded by 12 outer segments that assess GAC principles between 0.0 and 1.0. Color gradients are applied to illustrate each principle, ranging from red to yellow to green. Segment thicknesses designate the comparative importance of each principle. The Blue Applicability grade Index (BAGI) is also used to evaluate their practicality and applicability. The blue-filled region indicates the assessed categories, where dark blue represents the highest score, followed by light blue, and the unfilled white region indicates aspects that likely scored lower or were less favorable. In the ComplexGAPI assessment of the developed HPLC method, most segments were rated green for sample preparation, solvent usage, energy usage, and waste generation, confirming the method’s environmental safety and ease of operation.

## 3 Results

### 3.1 Optimization of the Developed Method

Initially, C18 column was chosen as a stationary phase carefully to maximize benefits. In this separation, it could overcome the drawbacks of normal phase columns, including the water hydration of silica, which is predicted to cause peaks to tail off. A C18 column (250 × 4.6 mm, 5 μm) offered an acceptable resolution as a result. The method was optimized using the OFAT (One factor at A Time) approach by systematically examining the effects of different organic solvents, such as methanol, and pH changes in phosphate buffers (Figure 1). Optimized RP-HPLC conditions comprised phosphate buffer (pH 3.0): methanol (95:5 *v/v*) at a flow rate of 0.6 mL/min and column temperature of 30 °C. This method provided better resolution with shorter retention times: ferrous fumarate (FF) – 8.5 ± 0.2 min and ascorbic acid (AA) – 6.4 ± 0.2 min at 220 and 245 nm detection, respectively. The key experimental factors influencing the separation process were identified and visualized using the fishbone diagram (Ishikawa diagram) (Figure 1).

**Figure 1:**
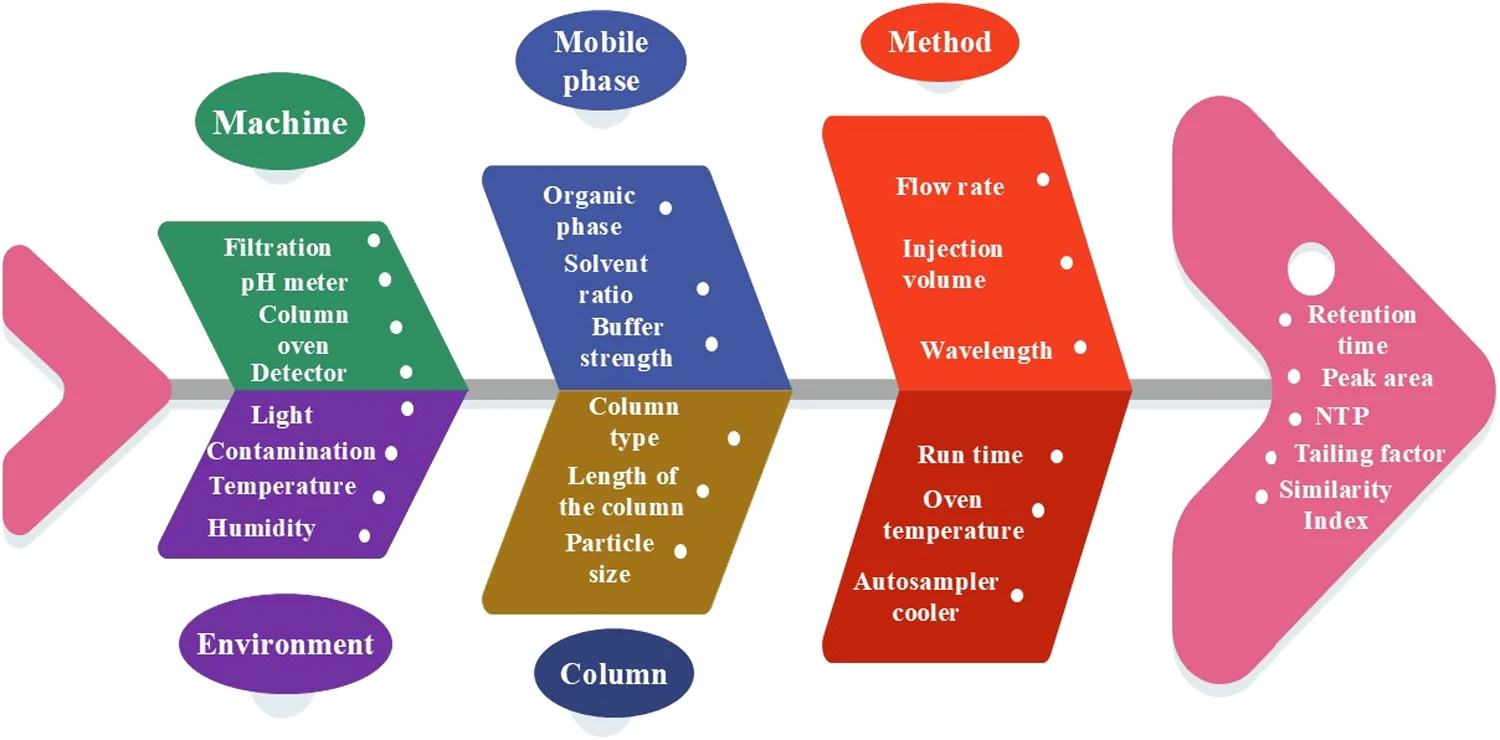
A visual tool (Ishikawa diagram) for identifying risk factors and root causes.

Ascorbic acid (AA) and ferrous fumarate (FF) eluted after approximately 6.4 and 8.5 min, respectively, under optimal conditions. Both drug molecules showed good solubility and stability when a higher proportion of phosphate buffer was added. An isocratic elution system is therefore required to properly retain both molecules using the optimal flow rate, since AA tends to elute with minimal retention. A validated isocratic elution system was optimized at a flow rate of 0.6 mL/min, using methanol and phosphate buffer at pH 3.0 (adjusted with orthophosphoric acid). Analysis run time was 12 min per sample at a constant 30 °C column temperature, yielding excellent resolution and minimal tailing for both molecules. PDA detector was used for simultaneous detection of samples at 220 and 245 nm. The system suitability parameters, namely, resolution ≥2, tailing factor ≤2, theoretical plates ≥2000, adequate sensitivity, and an acquisition time of 12 min, supported chromatographic performance.

Resolution, sensitivity, and speed were all satisfactory with this system. Figure 2 illustrates the chromatogram measured under optimized HPLC method conditions (at 220 nm for FF and 245 nm for AA).

**Figure 2:**
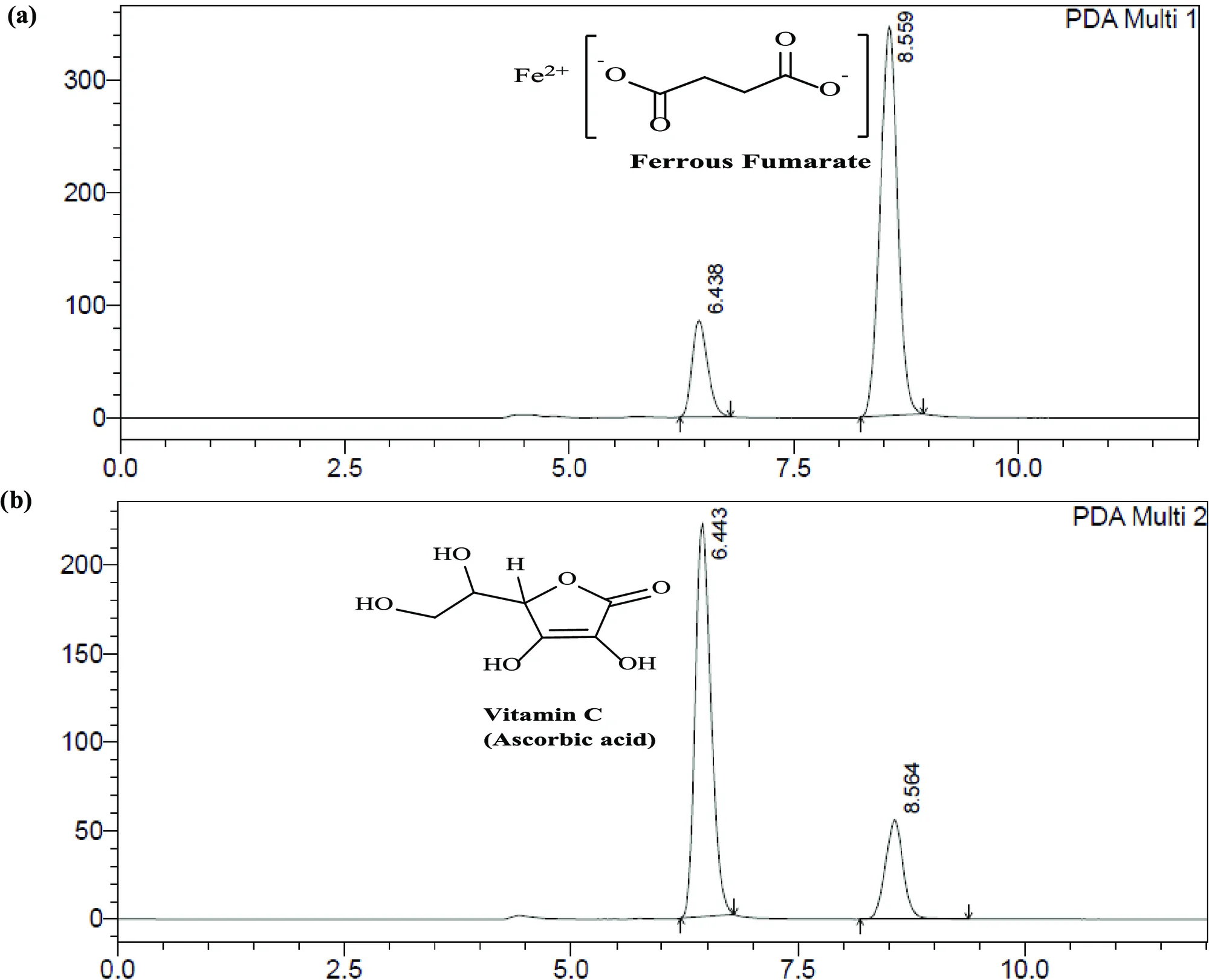
Chromatograms of Ferrous fumarate (FF) and Ascorbic acid (AA) at a flow rate of 0.6 mL/min. Chromatogram of ferrous fumarate (FF) shows maximum absorption at 220 nm (a), and (b) chromatogram of ascorbic acid (AA) shows maximum absorption at 245 nm.

### 3.2 Method Validation

A robust, validated HPLC method enables simultaneous FF and AA analysis, meeting system suitability, linearity, LOD, LOQ, and accuracy requirements. The RSD values of the validated parameters were computed and recorded to be within the acceptable range (<2% for analytical samples) (Table 2).

**Table 2:** Summary of Calibration, Linearity, and Sensitivity Parameters for the Simultaneous Estimation of Ferrous Fumarate and Ascorbic Acid

parameters	ascorbic acid (AA)	ferrous fumarate (FF)
range of calibration curve (μg/mL)	0.1–50	0.1–50
detection wavelength (nm)	245	220
retention time (minute)	6.4 ± 0.2	8.5 ± 0.2
correlation coefficient (*r* ^2^)	0.991	0.999
linearity equation	*y* = 82189*x*–178885	*y* = 93638*x*+75627
slope	82189	93638
intercept	178885	75627
LOD (μg/mL)	0.17	0.52
LOQ (μg/mL)	0.53	1.58

#### 3.2.1 System Suitability

Before validating the developed analytical method, system suitability must be assessed to avoid any system-induced disturbances. System suitability test for FF and AA was found acceptable, with a resolution greater than 6, a tailing factor less than 2, and a theoretical plate value greater than 5000. Both analytes were found to be stable at RT, with 6.4 ± 0.2 min (for AA) and 8.5 ± 0.2 min (for FF) across multiple injections at a concentration of 4 μg/mL (*n* = 6).

#### 3.2.2 Linearity, LOD and LOQ

A series of dilutions of FF and AA were analyzed to predict the linearity between their standard concentrations and average peak areas by plotting a calibration graph. The linear regression equations for FF and AA were found to be, *y* = 93638*x*+75627, *r* ^2^ = 0.999 and *y* = 82189*x*–178885, *r* ^2^ = 0.991, respectively (Figure 3), where “*x*” represents the concentration and “*y*” represents the peak area of the calibration curve of FF and AA, respectively. Further, the LOD and LOQ values for both molecules were computed mathematically in accordance with ICH guidelines, using the residual standard deviation (σ) and slope (S) of the calibration curve. Different regression analysis parameters for the AA and FF standard curves are mentioned. The LOD and LOQ values were 0.52 and 1.58 μg/mL for FF, and 0.17 and 0.53 μg/mL for AA, respectively (Table 2). In addition, a 3D chromatogram of FF and AA was also shown in Figure 3d.

**Figure 3:**
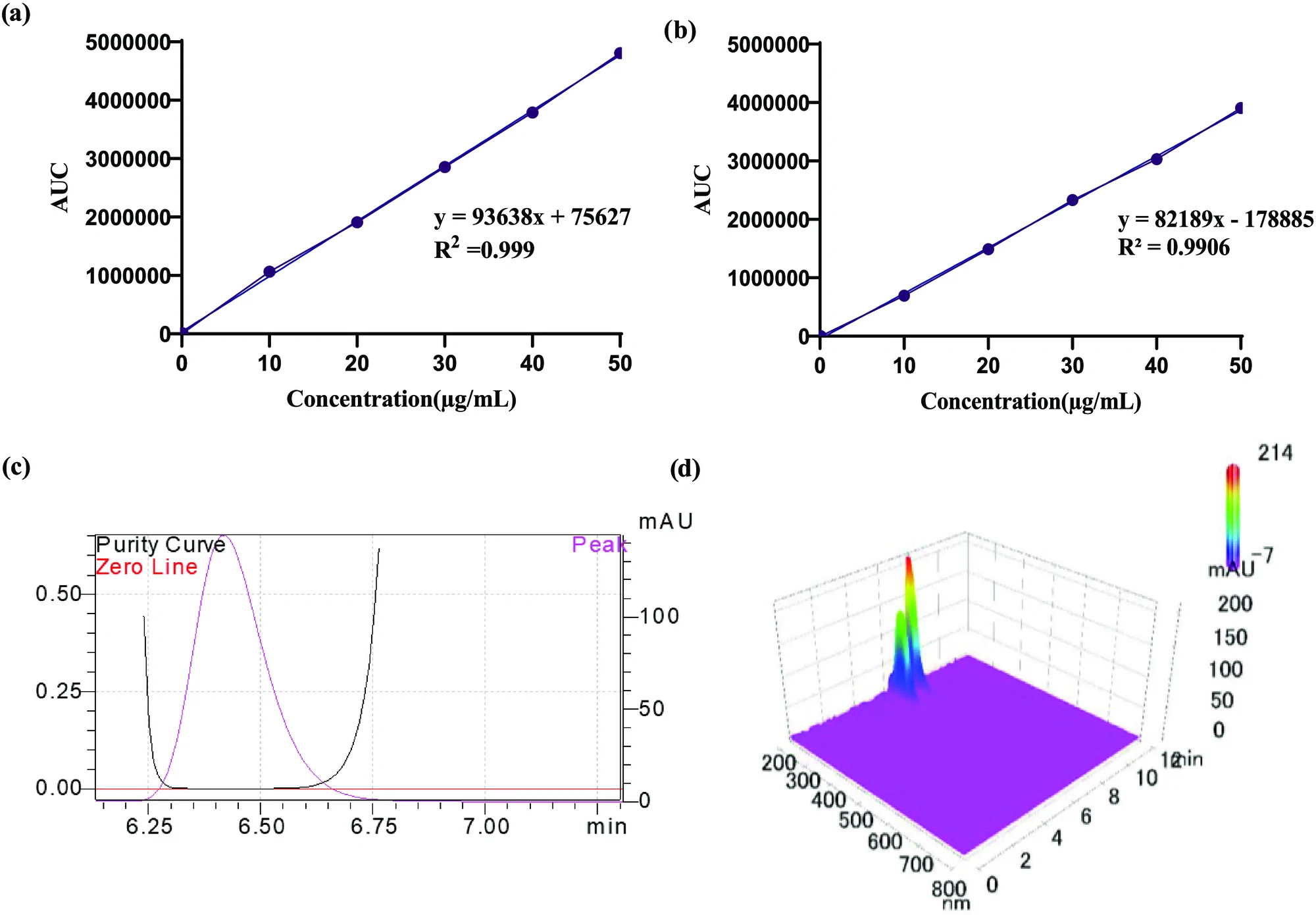
Calibration curves for (a) ferrous fumarate (FF) and (b) ascorbic acid (AA), (c) purity index, and (d) three-dimensional analyte plot, constructed using standard solutions (0.1 to 50 μg/mL; 6 levels × 3 replicates) via isocratic RP-HPLC.

#### 3.2.3 Accuracy and Precision

To assess the accuracy and precision of the validated method, the QC samples at lower, middle and higher levels, including 4, 5, and 6 μg/mL of both FF and AA, were assessed on the basis of their RSD values. The % recovery to estimate accuracy was noted within the standard acceptable range for both FF (98.46%) and AA (98.10%), justifying the accuracy of the developed method (Supporting Information; Table S1). Also, the % RSD values for FF and AA in the intraday and interday precision studies were less than 2%, confirming the precision of the developed method, as presented in Table 3.

**Table 3:** Precision Parameters of FF and AA

precision (FF)	intraday precision			interday precision		
	4 μg/mL	5 μg/mL	6 μg/mL	4 μg/mL	5 μg/mL	6 μg/mL
peak area (mean ± SD) (*n* = 3)	748227.5 ± 169.96	911563 ± 2168.05	1176753.3 ± 83.08	754639.25 ± 2315.14	913201 ± 3212.70	1180226.25 ± 2276.17
% RSD	0.69	0.23	0.63	0.30	0.35	0.19

precision (AA)	intraday precision			interday precision		
	4 μg/mL	5 μg/mL	6 μg/mL	4 μg/mL	5 μg/mL	6 μg/mL
peak area (mean ± SD) (*n* = 3)	5023.5 ± 89.45	73509.75 ± 997.24	192126.25 ± 3455.66	1630.75 ± 15.47	44176 ± 627.70	156920 ± 2030.30
% RSD	1.78	1.35	1.79	0.94	1.42	1.29

#### 3.2.4 Robustness

The robustness of the method was evaluated by introducing small deliberate variations in method parameters, including mobile phase composition (±2%), column temperature (±2 °C), and flow rate (±0.1 mL/min). The robustness study showed %RSD values within the acceptable limit of ≤2.0%, indicating that the deliberate variations in chromatographic conditions did not cause any significant change in peak area. However, slight shifts in retention time were noticed with changes in flow rate, likely due to altered analyte migration through the column.^36^ Overall, the method remained robust under all tested conditions (Table 4).

**Table 4:** Robustness Parameters of FF and AA

robustness FF					
parameter	level	retention time (min)		peak area (mV-min)	
		Mean ± SD (*n* = 3)	% RSD	mean ± SD (*n* = 3)	% RSD
ratio of buffer in mobile phase	93	7.30 ± 0.00	0.04	735799.25 ± 2171.50	0.29
	97	6.91 ± 0.00	0.11	395065.33 ± 4525.54	1.14
injection volume	18	7.92 ± 0.01	0.02	349781.33 ± 2285.34	0.58
	22	8.28 ± 0.01	0.16	997061.50 ± 3571.75	0.35
oven temperature	30	7.59 ± 0.00	0.04	1063713.50 ± 2235.61	0.21
	32	7.63 ± 0.00	0.05	362874.50 ± 3091.18	0.85
flow rate	0.5	9.60 ± 0.01	0.12	896014.75 ± 228.29	0.24
	0.7	7.45 ± 0.01	0.20	403727.00 ± 4263.42	1.05

robustness AA					
parameter	level	retention time (min)		peak area (mV-min)	
		mean ± SD (*n* = 3)	% RSD	mean ± SD (*n* = 3)	% RSD
ratio of buffer in mobile phase	93	4.43 ± 0.00	0.15	12366.33 ± 127.16	1.02
	97	4.48 ± 0.00	0.03	4966.00 ± 5.29	0.10
injection volume	18	4.42 ± 0.00	0.24	9533.66 ± 20.98	0.58
	22	4.43 ± 0.00	0.15	16371.00 ± 250.18	1.52
oven temperature	28	6.37 ± 0.00	0.05	605255.50 ± 1874.82	0.30
	32	6.43 ± 0.00	0.02	1279403.75 ± 8178.23	0.63
flow rate	0.5	4.43 ± 0.00	0.15	16371.00 ± 250.18	1.52
	0.7	4.48 ± 0.00	0.03	4966.00 ± 5.29	0.10tod

#### 3.2.5 Specificity

The optimized RP-HPLC method exhibited exceptional specificity, as no interfering peaks were observed at the retention times of ferrous fumarate, ascorbic acid, and blank chromatogram (Figure 4). The analyte peaks were well resolved, sharp, and free from coeluting signals, confirming that the method is specific for the simultaneous estimation of both drugs.

**Figure 4:**
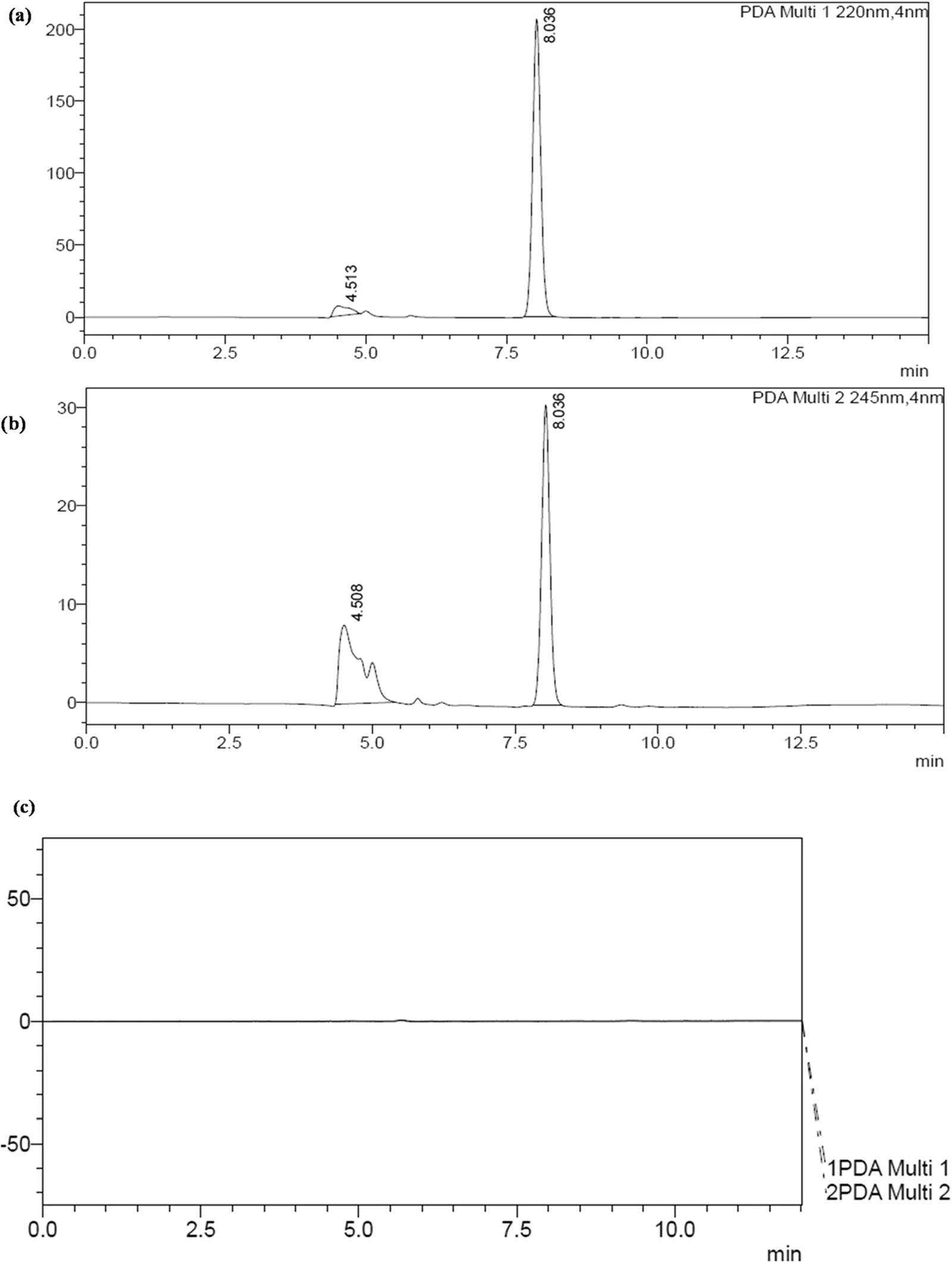
FF, AA and Blank Chromatograms (a) chromatogram of ferrous fumarate in the developed pro-liposome formulation; (b) chromatogram of ascorbic acid in the developed pro-liposome formulation; (c) blank chromatogram, obtained with mobile phase alone.

#### 3.2.6 Forced Degradation Study

The reliability testing of ascorbic acid (AA) and ferrous fumarate (FF) showed degradation peaks at retention times (RT) of 5.9 ± 0.01 and 7.8 ± 0.01 min for AA and FF, respectively, after treatment with 0.1 N HCl. These peaks are presumed to correspond to degradant products of FF and AA, as indicated by the shifted retention times. Under 0.1 N NaOH, no detectable degradation peak was observed for FF, while the degraded product of AA (most probably dehydroascorbic acid) might be retained at 4.79 min. Under 0.6% H_2_O_2_ stress conditions, AA might be degraded to dehydroascorbic acid with an RT of 5.9 min, and FF might also be degraded (RT: 7.8 min) due to the oxidation of ferrous to the ferric form (Figures S1–S3). In general, oxidative degradation of ascorbic acid might have proceeded through a two-electron oxidation to dehydroascorbic acid, which is consistent with the mechanism described by Deutsch.^37^

### 3.3 Applications of Method

#### 3.3.1 Characterization, Entrapment Efficiency and Drug Loading

The particle size, surface charge and polydispersity index (PDI) of the developed pro-liposomes were determined by using ZetaSizer (Malvern Panalytical Ltd., Malvern, UK). The particle size, ζ-potential, and the PDI of the pro-liposomes were found to be 183.3 nm, −39 mV, and 0.398, respectively. Entrapment efficiencies of 92% for FF and 65% for AA demonstrate that the formulation was highly effective for encapsulating both drugs, especially for ferrous fumarate. There may be greater partitioning into the lipid structure or a more favorable retention within the vesicular matrix for FF, while AA may be more difficult to retain because it is more hydrophilic and chemically labile. Therefore, it is evident that the pro-liposome system was able to protect and encapsulate a substantial portion of both these molecules. It was found that FF and AA pro-liposomes carried drug loading values of 40.23 and 6.35%, respectively. With the much higher loading of FF in comparison to AA, the formulation carried iron more efficiently, consistent with its higher entrapment efficiency. Additionally, this difference suggests that FF may be more easily incorporated into the formulation than AA (Figure 5).

**Figure 5:**
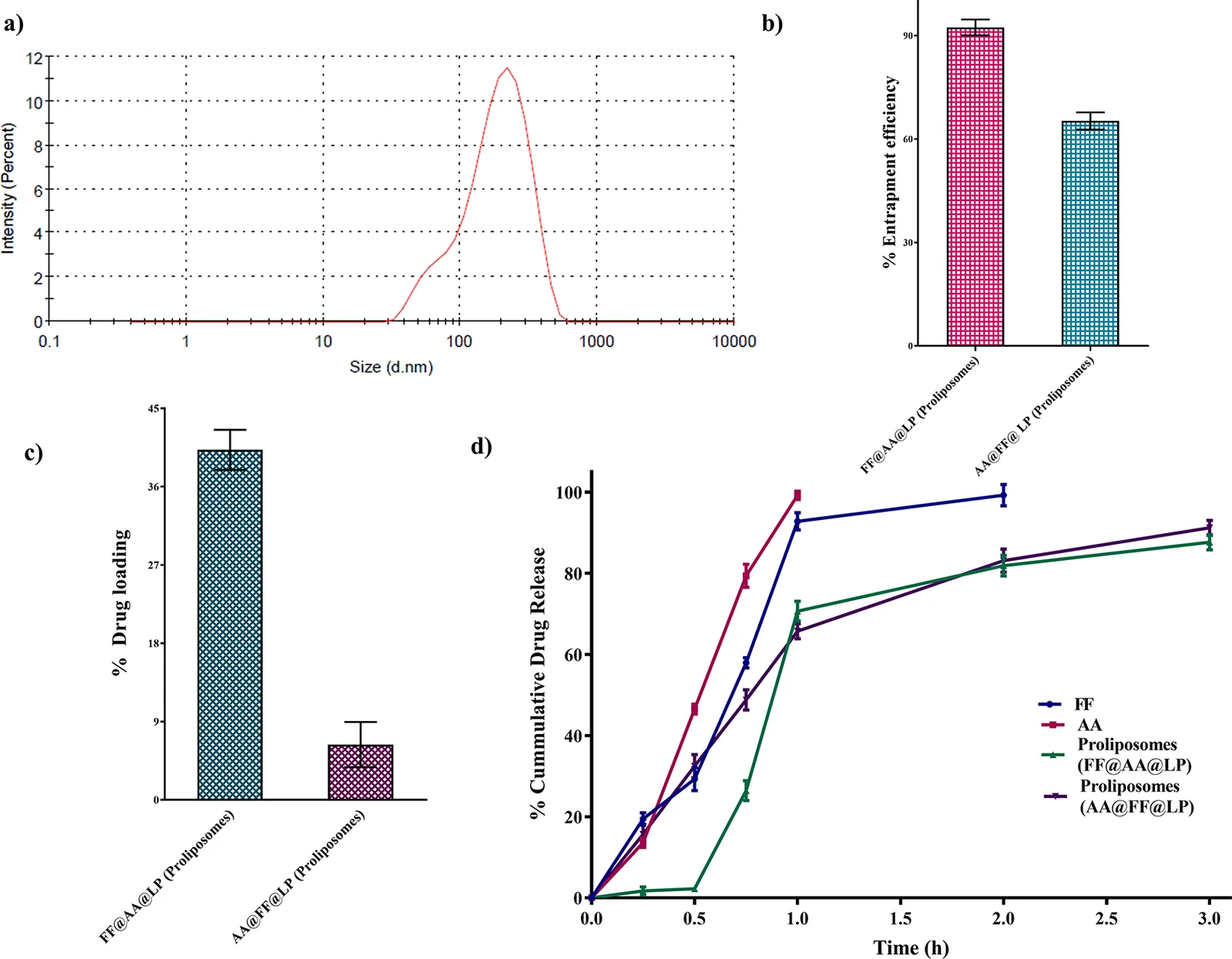
Formulation containing ferrous fumarate (FF) and ascorbic acid (AA) was comprehensively evaluated; (a) particle size distribution; (b) results of entrapment efficiency; (c) results of drug loading; (d) *in vitro* drug release profiles. Note: FF@AA@LP and AA@FF@LP terms were used to denote FF and AA, respectively, in the pro-liposomes in Entrapment Efficiency (%), Drug Loading and In Vitro Drug Release Study.

#### 3.3.2 *In Vitro* Drug Release Studies

The *in vitro* release profiles of FF and AA from the pro-liposomes were estimated using the dialysis bag method.^35,38^ FF release from pro-liposomes was 70.67% after 1h; whereas plain FF exhibited 92.84% release within 1 h at pH 1.2. Similarly, AA release from the pro-liposomes was 65.73%, whereas plain AA showed almost 100% (99.21%) release within 1 h at pH 1.2.^39^ Plain FF release was almost 100% (99.23%) within first 2 h at pH 1.2 The release of FF and AA from pro-liposomes at the end of 2 h at pH 1.2 was found to be 81.86 and 83.11%, respectively. At pH 4.5, AA release from pro-liposomes reached to 91.56% at the end of third hour, whereas FF release reached to 87.63% (Figure 5). The higher drug release of actives from pro-liposomes, though, was less than their plain form, could be because of the good solubility of actives in dissolution medium, and the smaller size of the pro-liposomes, which provided a large surface area.

### 3.4 Assessment of Greenness

A total value of 0.64 was obtained for the optimized method, indicating excellent environmental compatibility. Figure 6 illustrates the detailed score distribution for each GAC principle. High scores for minimizing sample volume, automation of analytical processes, avoiding derivatization, using safer reagents, and ensuring operator safety align with the principles of Green Analytical Chemistry. These results illustrate that the method is effective and resource-mindful, especially in terms of environment safety. A medium score for direct analysis, in situ measurements, and energy minimization suggests that sample preparation and some energy consumption remain part of the procedure. The minimum score for waste management and renewable solvent use highlights key areas needing improvement, as the method currently lacks recycling or biodegradable mobile phases. Overall, the method effectively aligns with sustainability principles but could be made greener through solvent replacement, waste reduction strategies, and enhanced energy efficiency. An AGREE pictogram dominated by green (score: 0.64) shows a red segment for offline sampling (common methodology limitation). Principles 6 and 9 score green through green solvents, rapid dual-analyte separation (<5 min), waste minimization, and operator safety improvements. The ComplexGAPI pictogram indicates that the corresponding criterion is in the good green category or has a minimum environmental burden, depending on the specific box and lower value. The BAGI assessment pictogram was obtained after evaluating factors such as analysis type, number of analytes, sample preparation, reagents, automation, and sample throughput.^40^ The results of 67.5 appear positive but not maximal, indicating that the method or sample was acceptable but could be improved. The graphical assessments indicate a reasonably good result, with balanced performance across most categories.

**Figure 6:**
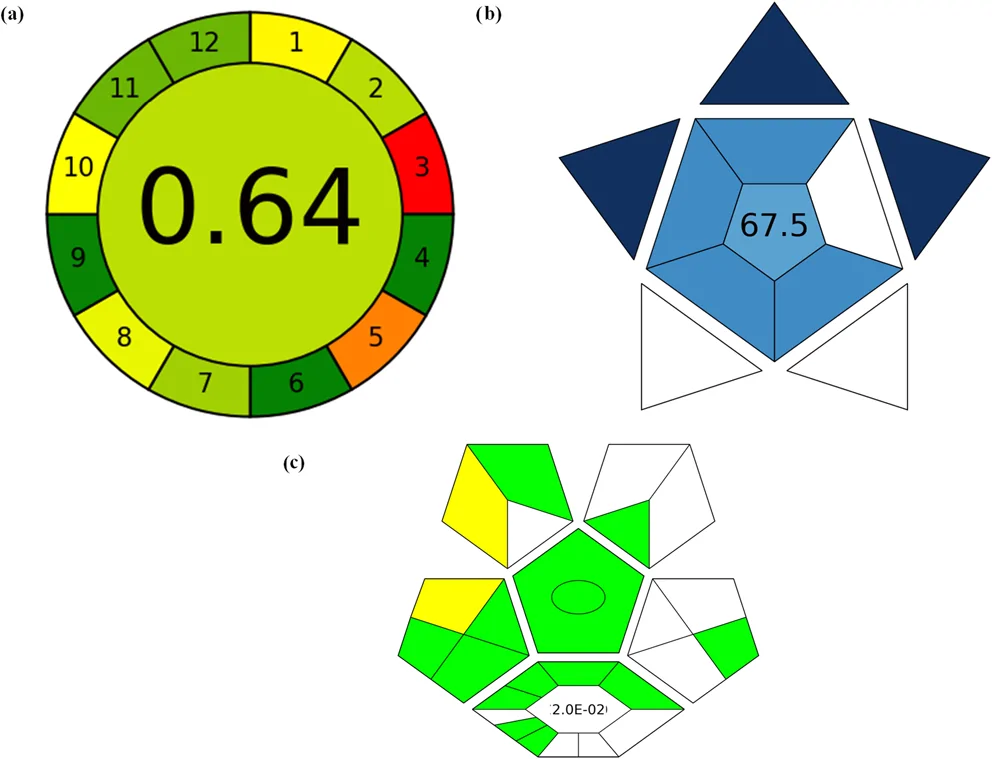
Green analytical assessment of the HPLC method: AGREEprep pictogram (a) showing overall score of 0.64 with all categories rated green. Contributions to optimal scores included manual sample preparation, eco-friendly phosphate buffers, simultaneous dual-analyte analysis (<8 min), minimal waste generation, and enhanced operator safety, particularly in Section 4, 6, and 9. Results were further supported by BAGI assessment (b) yielding a score of 67.5 and GAPI assessment pictogram (c).

## 4 Conclusion

In compliance with ICH Q2­(R1), an RP-HPLC-PDA method was developed and validated for the simultaneous analysis of ferrous fumarate (FF) and ascorbic acid (AA). For the simultaneous analysis of both molecules, the suggested approach presented good linearity, accuracy, selectivity, precision, and robustness. The reproducibility and robustness of the developed method were confirmed by high resolution, simultaneous elution of FF and AA within 10 min, and %RSD values less than 2. The validated method verified and tested for the quantitative analysis of FF and AA in both quality control samples and formulation, with no interference recorded from excipients or degradation. Additionally, the broad applicability of the method in formulation development, advancement and quality control examination was established by its use in analyzing *in vitro* release samples, drug loading and entrapment efficiency of cocktail molecules in pro-liposomes. In summary, the optimized HPLC method provides a rational approach to routinely estimating ferrous fumarate and ascorbic acid in pro-liposomes which may be applied to other pharmaceutical formulations. One of the limitations of this work is lack of a formal Design of Experiments (DoE) in the analytical method-development workflow. A planned DoE would cover a broader experimental range, such as alternative buffer systems and ionic strengths, different stationary phase chemistries, a wider range of organic modifiers, and long-term robustness across different HPLC platforms and column types. The optimized isocratic RP-HPLC method demonstrated eco-friendly, green, and sustainable characteristics.

## Supplementary Material



## Notes

Accuracy parameters for ferrous fumarate (FF) and ascorbic acid (AA) (Table S1); chromatograms of (a) ferrous fumarate (FF) and (b) ascorbic acid (AA) at 220 and 245 nm after exposure to 0.1 N HCl for 4 h under optimized conditions (Figure S1); chromatogram showing no detectable peak for ferrous fumarate (FF) and chromatogram of ascorbic acid (AA) after exposure to 0.1 N NaOH for 2 h under optimized conditions (Figure S2); and chromatograms of (a) ferrous fumarate (FF) at 220 nm and (b) ascorbic acid (AA) (Figure S3) (PDF)
