# Supplementary material for: Development and Validation of a Simultaneous RP-HPLC Method for the Estimation of Ferrous Fumarate and Ascorbic Acid for Pharmaceutical Quality Control
Source: ACS Omega. 2026 Aug 18;11(34):50922–32. doi: 10.1021/acsomega.6c02561 (PMC13624945; doi:10.1021/acsomega.6c02561)
Supplement: Supplementary file 1 [file ao6c02561_si_001.pdf]

Development and Validation of a Simultaneous RP-HPLC Method for Estimation of Ferrous Fumarate and Ascorbic Acid for Pharmaceutical Quality Control

Vinay Kumar[1], Muralidhar Pisay[1], Anoushka Mukharya[1], Komal Rana[2], Sanjay Bharati[3], Muddukrishna Badamane Sathyanarayana[4], Srinivas Mutalik[1*]

[1] Department of Pharmaceutics, Manipal College of Pharmaceutical Sciences, Manipal Academy of Higher Education, Manipal 576104, Karnataka, India

[2] Manipal - Government of Karnataka Bioincubator, Advanced Research Centre, Manipal Academy of Higher Education, Manipal 576104, Karnataka, India

[3] Department of Nuclear Medicine, Manipal College of Health Professions, Manipal Academy of Higher Education, Manipal 576104, Karnataka, India

[4] Department of Pharmaceutical Quality Assurance, Manipal College of Pharmaceutical Sciences, Manipal Academy of Higher Education, Manipal 576104, Karnataka, India

* Corresponding author:

Srinivas Mutalik

Professor and Principal

Manipal College of Pharmaceutical Sciences

Manipal Academy of Higher Education

Manipal 576104, Karnataka, India

Email: ss.mutalik@manipal.edu

Table S1: Accuracy parameter of FF and AA

% Accuracy FF				% Accuracy AA			
**Concentration Level (%)**	**Initial Concentration (µg/mL)**	**Observed Concentration (µg/mL)**	**% Mean Recovery**	**Concentration Level (%)**	**Initial Concentration (µg/mL)**	**Observed Concentration (µg/mL)**	**% Mean Recovery**
80% (Low)	4.00	3.94	98.50	80% (Low)	4.00	3.98	99.50
100% (Medium)	5.00	4.96	99.20	100% (Medium)	5.00	4.79	95.80
120% (High)	6.00	5.86	97.67	120% (High)	6.00	5.94	99.00
**Average % Recovery**			98.46	**Average % Recovery**			98.10
**% RSD**			0.53	**% RSD**			1.99

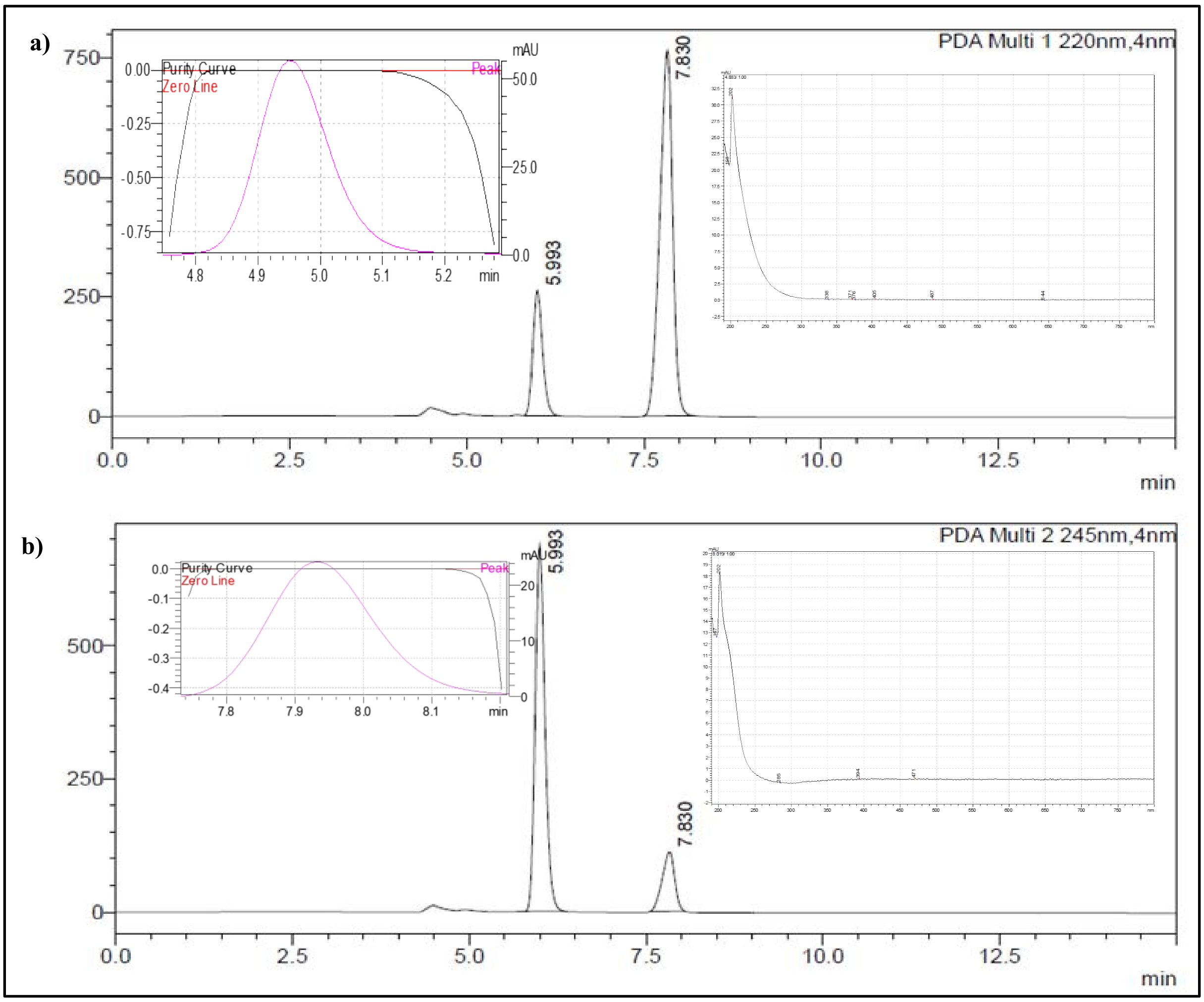

Figure S1: Chromatograms of (a) ferrous fumarate (FF) and (b) ascorbic acid (AA) at 220 nm and 245 nm after exposure to 0.1 N HCl for 4 h under optimized conditions.

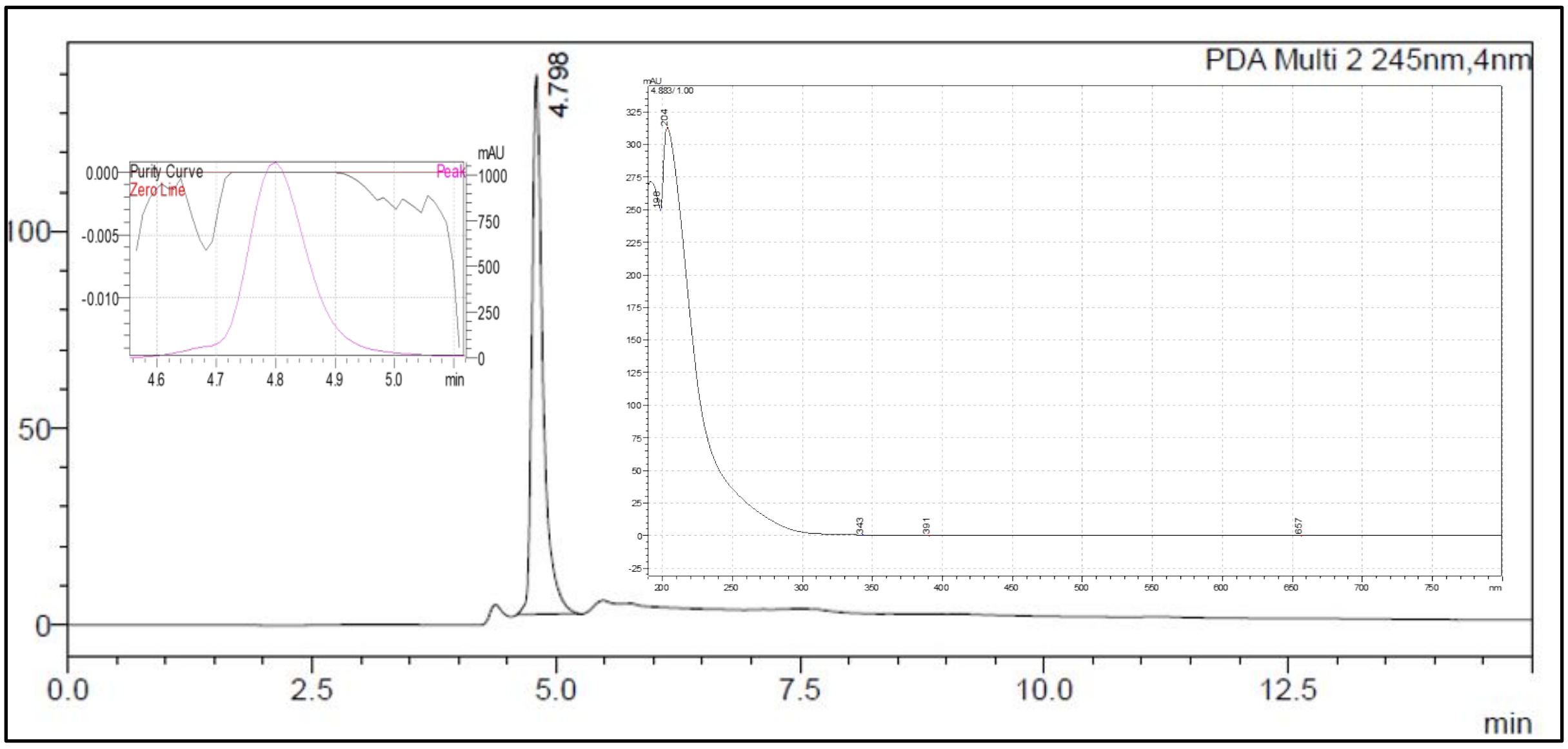

Figure S2: Chromatogram of ferrous fumarate (FF) (no peak detected) and ascorbic acid (AA) after exposure to 0.1 N NaOH for 2 h under optimized conditions.

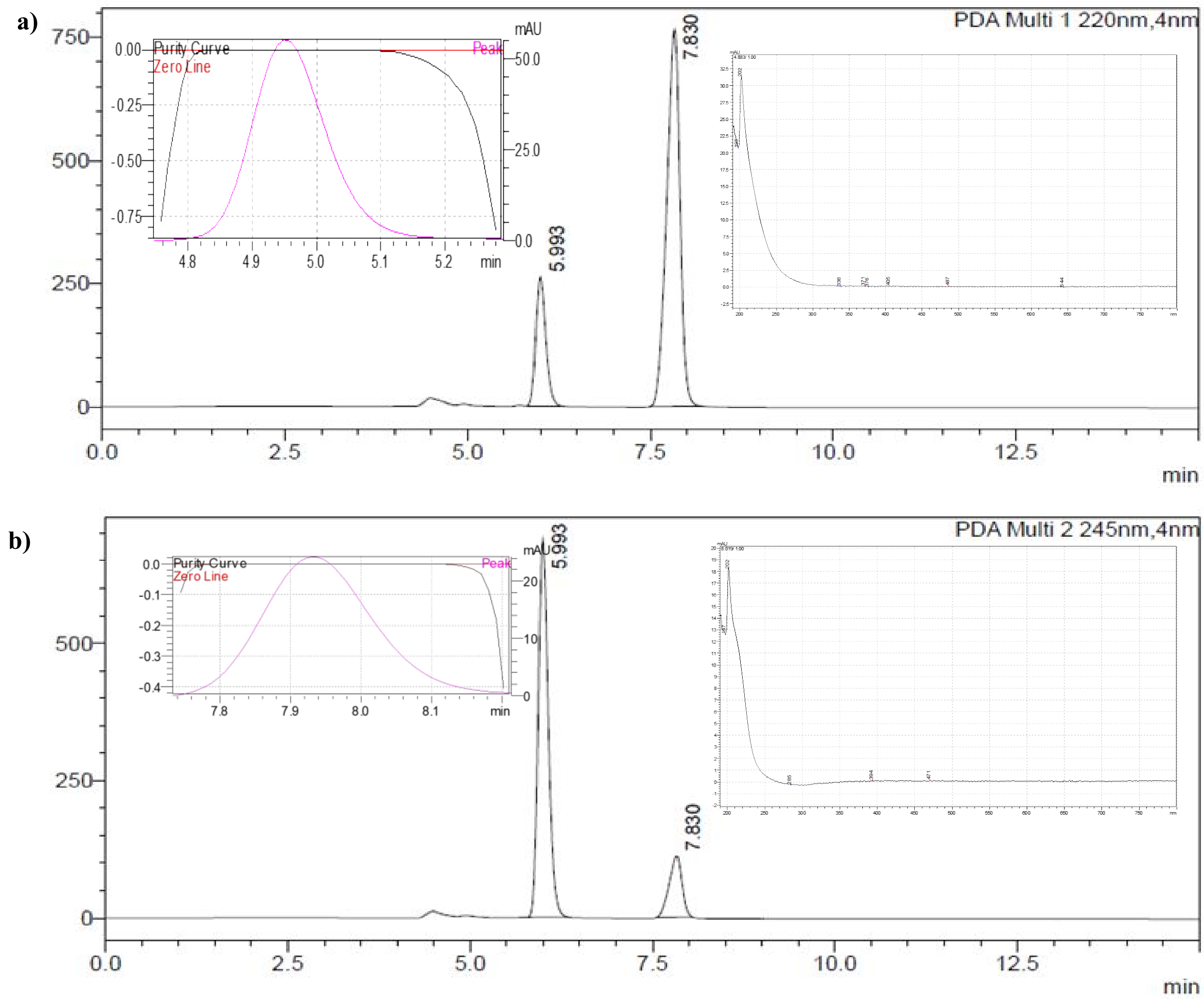

Figure S3. Chromatograms of (a) ferrous fumarate (FF) at 220 nm and (b) ascorbic acid (AA) at 245 nm after exposure to 0.6% H_2O_2, under optimized conditions.
